# Identification of hub genes and potential molecular mechanisms related to radiotherapy in thyroid cancer

**DOI:** 10.1097/MD.0000000000041140

**Published:** 2025-01-03

**Authors:** Chun-Tao Liao, Xing-Feng Tu, Guo-Liang Lin, De-Jie Zhang, Peng-Fei Li, Ming Zhang

**Affiliations:** a Thyroid and Breast Surgery, Longyan First Affiliated Hospital of Fujian Medical University, Longyan, Fujian, China.

**Keywords:** nomogram, prognosis, radiotherapy, thyroid cancer

## Abstract

Radiotherapy is a common approach during the treatment of thyroid cancer (THCA). It is urgent to identify the radiotherapy-related gene and explore the underlying mechanisms. An message RNA expression clinical data was gained from the Cancer Genome Atlas. The differential expression genes between normal individuals and THCA patients were identified by the “limma” package of R software. The differential expression genes between the patients without radiation therapy and the patients with radiation therapy were also obtained via the same method. Survival analysis, gene set enrichment analysis, immune analysis, drug sensitivity analysis, gene–miRNA, and nomogram analysis were performed to explore the radiotherapy-related gene value. The results showed that 354 DGEs between the THCA patients without radiation therapy and THCA patients with radiation therapy including the 148 up-regulated genes and 206 down-regulated were screened and displayed by volcano plot. A gene enrichment analysis showed radiation-related genes were enriched in various pathways such as mineral absorption, complement and coagulation cascades, B cell receptor signaling pathway, salivary secretion, and hematopoietic cell lineage. Then the hub-related-radiotherapy prognosis gene LRP1B was identified. The expression analysis showed that the LRP1B expression level was higher in normal individuals than in THCA patients with an obvious difference via *T* test in independent samples and paired samples. Immune analysis results showed that the stroma score, immune score, and ESTIMATES score were higher in the low-risk score than in the high-risk score. LRP1B is a vital gene that executes function via a variety of pathways in THCA patients with radiotherapy. Radiotherapy could reduce the expression of LRP1B and AL356596.1. Moreover, the constructed nomogram is based on risk score and clinical features, and it had a great function in predicting survival time for patients.

## 1. Introduction

Thyroid cancer (THCA) is the most common endocrine malignancy in the world. New epidemiological data indicated that there were 586,000 cases of THCA patients based on the Global Cancer Statistics 2020.^[1]^ Moreover, its incidence is increasing annually. In the past 30 years, its incidence has increased by 22 times in women and has increased by 15 times in men. It has become 1 of the top 10 cancers that threaten people’s health.^[2]^ THCA commonly was divided into 4 subtypes based on histological characteristics including papillary thyroid carcinoma, follicular thyroid carcinoma, medullary thyroid carcinoma, and anaplastic thyroid cancer. The first 2 are known as differentiated thyroid cancer (DTC) because of their high level of differentiation.^[3]^

Radiotherapy is widely and predominantly used treatment for patients with cancer. Radiation had the function of damaging DNA double-strand breaks in tumor cells, thereby inhibiting cancer cell proliferation. Moreover, it also directly kills cancer cells by ionizing radiation.^[4,5]^ Although the preferred treatment for DTC is surgery, radiotherapy is also often used in clinical. The development of radiation physics techniques with external radiation therapy, especially the implementation of intensity-modulated radiation therapy, has led to an increasing role for radiotherapy in the treatment of poorly DTC with poor or residual surgical margins and extensive lymph node metastases, especially in the treatment of thyroid cancer without 131I uptake. However, most studies focused on the effect of radiotherapy on treatment and indicated that postoperative adjuvant radiotherapy can improve the success rate of local treatment.^[6]^ However, research about radiotherapy-related genes was rare in THCA.

In this work, we screened the radiotherapy-related gene in THCA patients. The potential molecular mechanisms of radiotherapy effect in patients with THCA were explored via various bioinformatics methods. We found that the expression of the radiotherapy-related prognosis gene, LRP1B, was decreased via radiation treatments, and its low expression was beneficial to patient survival by various pathways.

## 2. Materials and methods

### 2.1. Data source

The raw message RNA (mRNA) expression file and clinical characteristic data were collected from the Cancer Genome Atlas-THCA dataset including 48 normal individuals and 502 THCA patients. And there were 235 patients with exact radiotherapy information including 78 patients without radiation and 157 patients without radiation. The mRNA expression data were normalized by the “limma” package.

### 2.2. Differently expressed genes identification

The differential expression genes (DEG) between the normal individuals and THCA patients were identified by the “limma” package of R software based on *P* < .05 and |log2 (fold change) | > 1. In addition, the DEGs between the patients without radiation therapy and the patients with radiation therapy were obtained via the same method. The common genes between the 2 DEG sets were screened via the “Venn” package of R software.

### 2.3. Functional enrichment analysis

To further explore the function of DEGs, the “clusterProfiler” R package 15 was employed to perform KEGG analyses. *P*-values were adjusted by the Bebjamini and Hochberg method. The Gene Ontology function enrichment analysis was also performed based on the DEGs, and cellular component, biological process, and molecular function were annotated. Moreover, the condition of significant pathways included nominal *P*-value < .05, false discovery rate q-value < 0.25, and absolute normalized enrichment score > 1.

### 2.4. Survival analysis

Kaplan–Meier curves were constructed according to overall survival (OS) and the gene expression of patients, while the optimal cutoff point for survival curves was generated through the “res. cut” function in the “survminer” package and the log-rank test was used to analyze the differences between survival curves. Additionally, the univariate and multivariable Cox regression analyses were used for calculating the association between the expression of DGEs and the patient’s OS to further screen the hub genes in patients with radiation therapy.

### 2.5. Gene set enrichment analysis

The Gene Set Enrichment Analysis (GSEA) was the first to rate an ordered list of DEGs. The GSEA was carried out to analyze the significant survival difference observed between the high-risk score and the low-risk score group of patients with radiation therapy. The dataset of c2.cp.kegg.v7.4.symbols.gmt was downloaded from the molecular signatures database and was used to evaluate related pathways and molecular mechanisms. Set permutations were performed 1000 times for each analysis. *P*-value of < .05 and a false discovery rate of < 0.25 were considered statistically significant.

### 2.6. Immune analysis

The microenvironment estimation was explored by the “ESTIMATE” package of the R software based on the mRNA expression data. The stromal score, immune score, and tumor purity of each patient with radiation therapy were obtained. The abundance of 22 kinds of tumor-infiltrating immune cells in THCA patients with radiation therapy was calculated via the “CIBERSORT” algorithm in the “CIBERSORT” package of R software.^[7]^ In addition, they were divided into 2 groups according to the risk score to explore the relationship between the hub genes and the immune microenvironment score of 22 kinds of immune cells.

### 2.7. Drug sensitivity analysis

To find the potentially available drugs, a drug analysis was performed via the website of the Gene Set Cancer Analysis (http://bioinfo.life.hust.edu.cn/GSCA/#/). The website is a public online platform for anticancer drug sensitivity and pharmacogenomic analysis.

### 2.8. Construction of Gene–miRNA–transcription factors interaction network

The Network Analyst (https://www.networkanalyst.ca/, June 11, 2022) database commonly is used to explore the correction between the gene and genes–transcription factors (TF) or miRNA. Gene–miRNA interaction database includes the miRtarBase v8.0, TarBase v8.0, and miRecords. Gene–TF interaction database includes ENCODE, JASPAR, and ChEA.

### 2.9. Construction of nomogram model

The univariate and multivariable Cox regression analyses were used for exploring the relationship between the expression of DEG and the patient’s OS to further determine hub genes in patients with radiation therapy. Based on the Cox regression results, a risk model based on hub genes was constructed. Then the R package “rms” was utilized to build the nomogram which integrated the risk model and clinical data of patients. The clinical feature was screened also by univariate and multivariable Cox regression analyses. Calibration curves and receiver operating characteristic (ROC) curves were constructed to evaluate the predictive performance of the nomogram model.

### 2.10. Statistical analysis

We used R software and SPSS version 21.0 software (IBM SPSS, Armonk, NY) to analyze the data in this work. A *T* test was used in the comparison of continuous variables in the 2 groups. The relationship analysis between the immune checkpoints and the expression of the hub gene via the Pearson analysis method. *P*-values < .05 were considered statistically significant.

## 3. Results

### 3.1. Screening of the prognosis radiation-related genes

**Figure 1.**
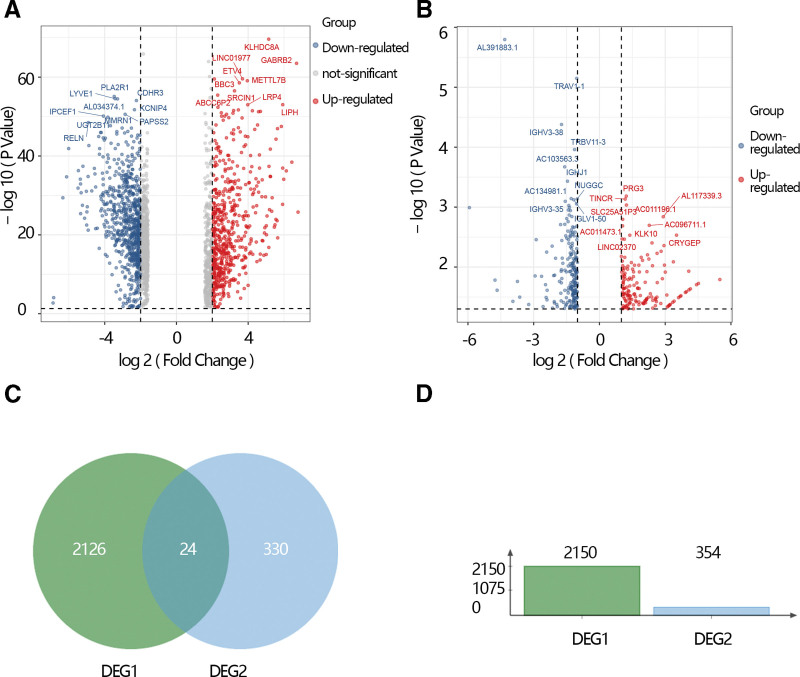
(A) Volcano plot of differential expression genes (DEGs) between the THCA patients and normal individuals. (B) Volcano plot of DEG between THCA patients with radiation therapy and THCA patients without radiation therapy. (C) Venn plot of the 2 DEGs. (D) The bar chart of 2 DEGs. DEG1 represents the DEG between THCA patients and normal individuals. DEG2 represents the DEG2 between THCA patients with radiation therapy and THCA patients without radiation therapy. THCA = thyroid cancer.

The DGEs analysis was performed based on the Cancer Genome Atlas database by the “limma” algorithm and the results showed that 2150 DGEs including 875 up-regulated genes and 1275 down-regulated genes were screened. DGEs were displayed by volcano plot (Fig. 1A). Meanwhile, the DGEs between the THCA patients without radiation therapy and THCA patients with radiation therapy. The results showed that 354 DGEs including the 148 up-regulated genes and 206 down-regulated and displayed by volcano plot (Fig. 1B). We obtained overlapped 24 genes by the Venn gram (Fig. 1C). Moreover, the histogram shows the number of 2 DGEs (Fig. 1D).

### 3.2. The gene enrichment analysis of radiation-related genes

**Figure 2.**
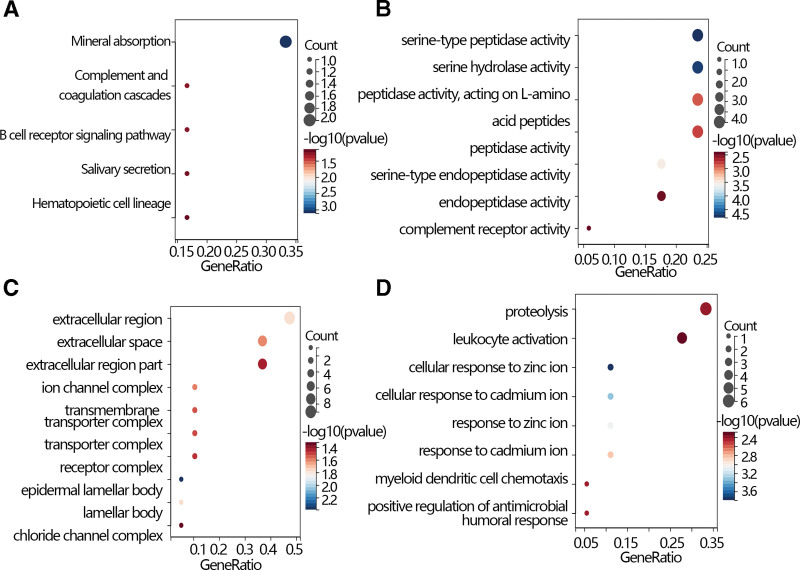
(A) The KEGG gene enrichment of overlapped genes between DEG1 and DEG2. (B) The GO analysis of the overlapped genes between DEG1 and DEG2 in molecular function, and (C) cellular component, (D) biological process. DEG = differential expression genes, GO = gene ontology.

A gene enrichment analysis was performed to further explore the function of radiation-related genes. The results showed that those genes were enriched in various pathways such as mineral absorption, complement and coagulation cascades, B cell receptor signaling pathway, salivary secretion, and hematopoietic cell lineage based on the KEGG database (Fig. 2A). Moreover, the Gene Ontology analysis showed that those genes enriched in the extracellular region, extracellular space, extracellular region part, ion channel complex, transmembrane, transporter complex, transporter complex, receptor complex, epidermal lamellar body, lamellar body, chloride channel complex in cellular component (Fig. 2C), involved in serine-type peptidase activity, serine hydrolase activity, peptidase activity, acting on L-amino, acid peptides, peptidase activity, serine-type endopeptidase activity, endopeptidase activity, complement receptor activity in molecular function (Fig. 2B), and related to proteolysis, leukocyte activation, cellular response to zinc ion, cellular response to cadmium ion, response to zinc ion, response to cadmium ion, myeloid dendritic cell chemotaxis, positive regulation of antimicrobial, humoral response in biological process (Fig. 2D).

### 3.3. Screening of the hub prognosis radiation-related genes

**Figure 3.**
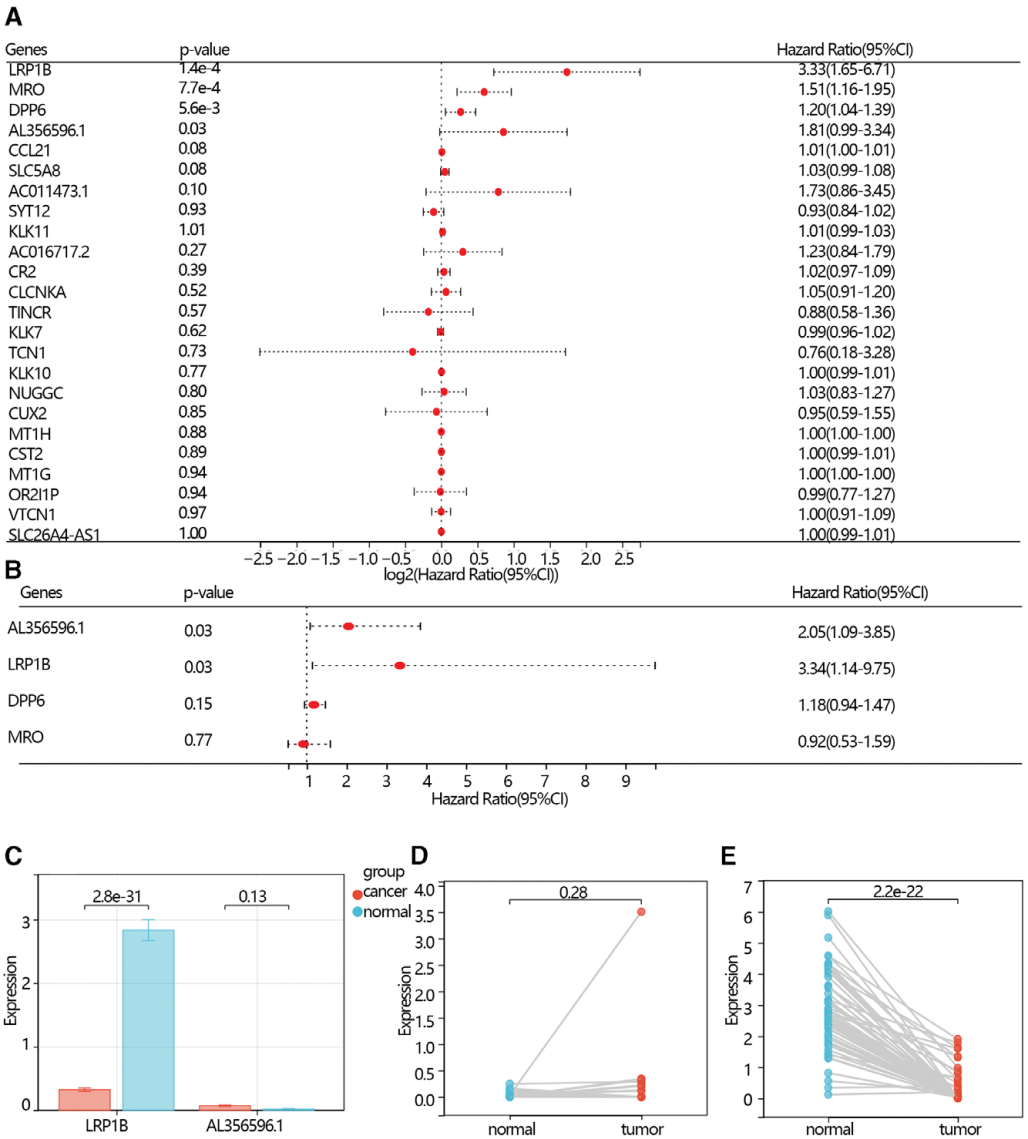
(A) The forest map of the univariate Cox regression analysis of overlapped genes in THCA patients. (B) The forest map of the multivariate Cox regression analysis of key genes. (C) The comparison of the hub gene between the normal group and the THCA group. (D) Paired test of AL356596.1. (E) Paired test of LRP1B. THCA = thyroid cancer.

The univariable Cox regression analysis was performed to screen the hub gene further. The results showed that LRP1B, MRO, DPP6, and AL356596.1 were screened according to the criteria of *P* < .05 (Fig. 3A). Then, these 4 genes were further included in a multivariate COX regression analysis, and the LRP1B and AL356596.1 were screened and served as the independent prognostic factors (Fig. 3B).

### 3.4. The expression analysis and survival analysis of the hub prognosis radiation-related genes

**Figure 4.**
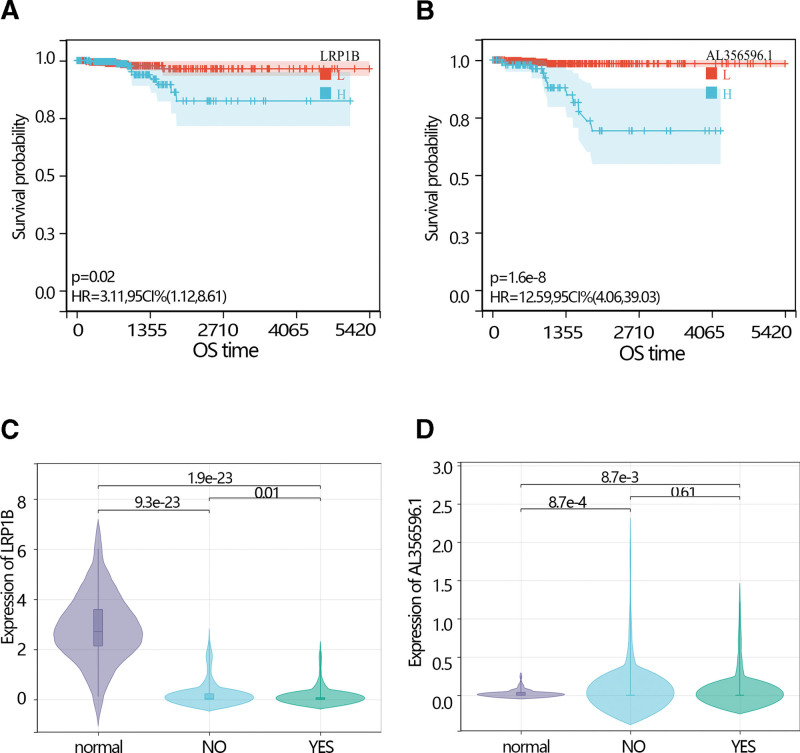
(A) The K–M plot of the expression of the LRP1B in THCA patients. (B) The K–M plot of the expression of the AL356596.1 in THCA patients. (C) The comparison of the expression of LRPIB among normal individuals, THCA patients without radiation therapy, and THCA patients with radiation therapy. (D) The comparison of the expression of AL356596.1 among normal individuals, THCA patients without radiation therapy, and THCA patients with radiation therapy. NO represents THCA patients without a radiation therapy group. YES represents THCA patients with a radiation therapy group. K–M = Kaplan–Meier, THCA = thyroid cancer.

**Figure 5.**
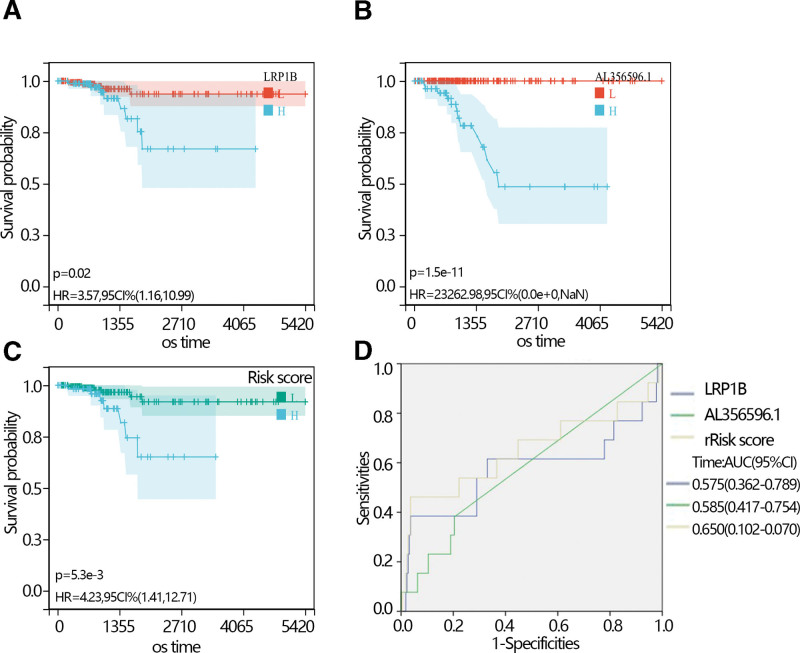
(A) The K–M plot of the expression of the LRP1B in THCA patients with accurate radiation therapy information. (B) The K–M plot of the expression of AL356596.1 in THCA patients with accurate radiation therapy information. (C) The K–M plot of the risk score in THCA patients with accurate radiation therapy information. (D) The ROC plot of LRP1B, AL356596.1, and risk score. K–M = Kaplan–Meier, ROC = receiver operating characteristic, THCA = thyroid cancer.

The expression analysis showed that the LRP1B expression level was higher in normal individuals than in THCA patients with an obvious difference via *T* test in independent samples and paired samples. However, the AL356596.1 expression level was lower in normal individuals than in THCA patients with an obvious difference via *T* test in independent samples and paired samples (Fig. 3C–E). The survival analysis showed that low expression of LRP1B and AL356596.1 favored the prognosis of THCA patients (Fig. 4A and B). The expression of LRP1B in the 3 groups was significantly different. Its expression in THCA patients with radiation was lower than that in THCA patients without radiation, and lower than in normal individuals (Fig. 4C). The expression of AL356596.1 in THCA patients with radiation was also lower than that in THCA patients without radiation, and lower than that in normal individuals (Fig. 4D). The results indicated that radiation reduced the expression of 2 genes. A survival analysis was performed to explore the prognosis of 2 genes in THCA patients with radiation. The results showed that low expression of LRP1B and AL356596.1 favored the prognosis of patients (Fig. 5A and B). Moreover, risk scores were calculated according to the gene expression and multivariate COX regression coefficient. The results showed that low-risk scores favored the prognosis of THCA patients (Fig. 5C). In addition, the ROC indicated that the risk score had a better prediction ability than LRP1B and AL356596..1 (Fig. 5D).

### 3.5 . The relationship between the hub gene and differentiation score

**Figure 6.**
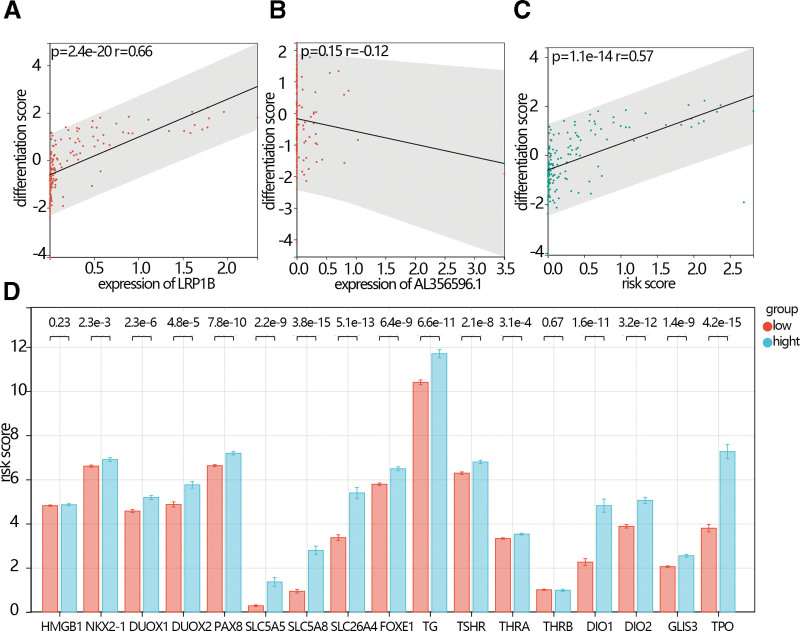
(A) The correlation between the differentiation score and expression of LRP1B. (B) The correlation between the differentiation score and expression of AL356596.1. (C) The correlation between the differentiation score and risk score. (D) The comparison of the differentiation score gene markers between the low-risk score group and the high-risk score group.

The differentiation is an important phenotype in THCA. We explored the relationship between the hub genes expression or risk score and differentiation. The results showed that the differentiation score was positively related to the expression of LRP1B (Fig, 6A, *P* = 2.4e-20, *R* = 0.66), while the differentiation score was negatively related to the expression of AL356596.1 (Fig. 6B, *P* = .15, *R* = 0.12). The differentiation score was positively related to risk scores (Fig. 6C, *P* = 1.1e-14, *R* = 0.57). The expression of differentiation-related gene markers including NKX2-1, DUOX1, DUOX2, PAX8, SLC5A5, SLC5A8, SLC26A4, FOXE1, TG, TSHR, THRA, DIO1, DIO2, GLIS3, and TPO was lower in the low-risk score group than they in the high-risk score group (Fig. 6D).

### 3.6. GSEA

**Figure 7.**
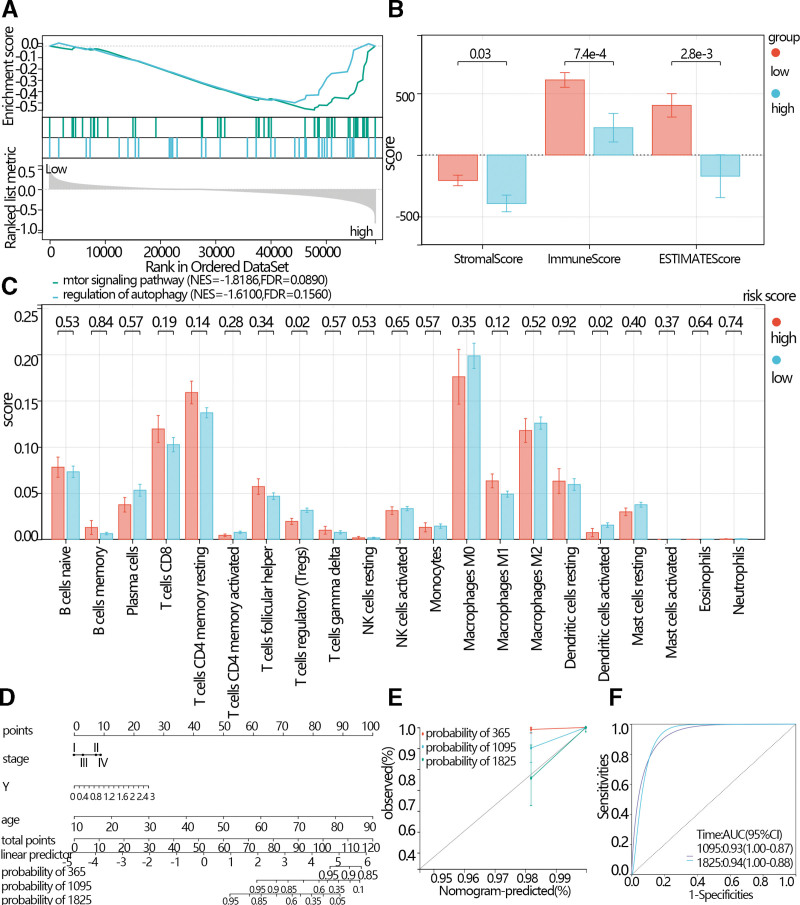
(A) Enrichment of pathways by GSEA. (B) The comparison of the stomal score immune score and ESTIMATES score between the low-risk score group and the high-risk score group. (C) The comparison of the immune cells’ abundance between the low-risk score group and the high-risk score group. (D) The Nomogram for prediction of the outcome of THCA patients. (E) The calibration curve. (F) ROC curve. GSEA = Gene Set Enrichment Analysis, ROC = receiver operating characteristic, THCA = thyroid cancer.

To explore the signaling pathways potentially related to risk scores, the GSEA was performed based on the risk scores group. According to the NES and normal *P* < .05, the key signaling pathways were screened. The results showed that the mTOR signaling pathway and regulation of autophagy were enriched in the high-risk score group (Fig. 7A).

### 3.7. Immune infiltration analysis

According to the results of the GSEA, the screened signaling pathways were related to the immune process. Therefore, immune infiltration analyses were performed. The results showed that the stroma score, immune score, and ESTIMATES score were higher in the low-risk score than in the high-risk score (Fig. 7B). The results indicated that the infiltration of T cell regulatory and dendritic cells activated in the low-risk score group was higher than in the high-risk score (Fig. 7C).

### 3.8. Construction of Nomogram

The univariate and multivariate regression analysis was performed based on the clinical features, expression of the hub gene, and risk score. The independence features were used to construct Nomogram. The C-index of the Nomogram was 0.93, (Fig. 7D, 95% CI: 0.89–0.98, *P*-value = 6.35e-87). And the calibration curve and ROC curve indicated that the prediction model had good prediction efficiency (Fig. 7E and F).

### 3.9. Drug sensitivity analysis

**Figure 8.**
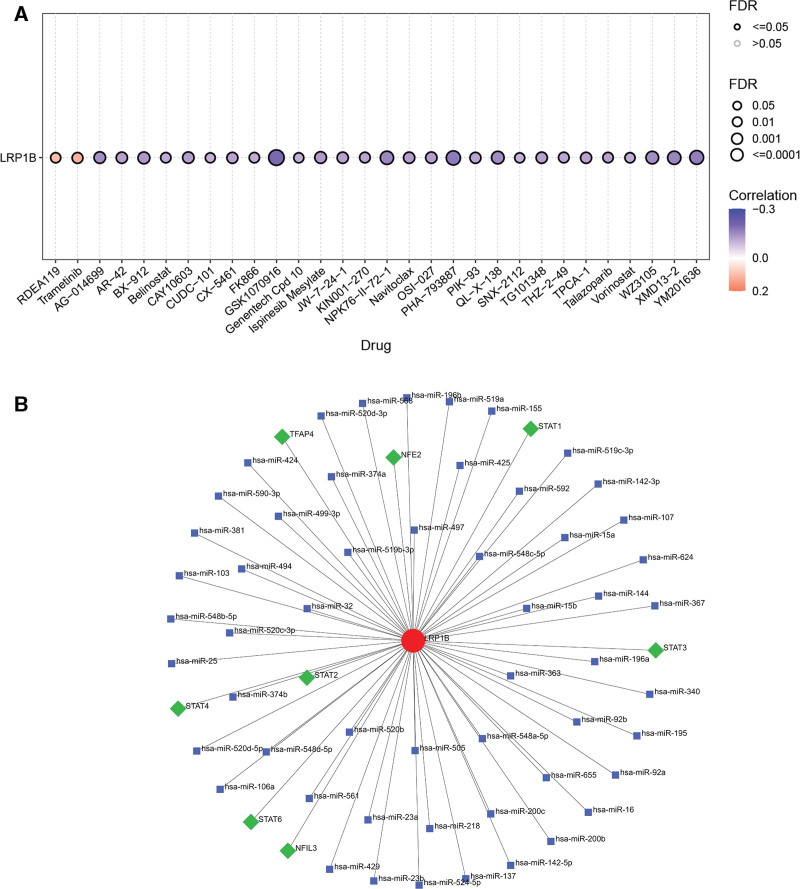
(A) Correlation between drug sensitivity and mRNA expression. (B) The interaction network of TF and miRNA drug. mRNA = message RNA, TF = transcription factors.

The drug sensitivity showed that the expression of the LRP1B was negatively related to many kinds of drugs such as AG − 014699, AR − 42, BX − 912, Belinostat, CAY10603, CUDC − 101, CX − 5461, and so on while was negatively related to RDEA119 and trametinib (Fig. 8A).

### 3.10. Results of gene–miRNA–TF

Gene and miRNA or TF interaction networks were constructed. The results showed that the LRP1B had 8 TFs including STAT2, STAT4, STAT6, NFIL3, STAT3, STAT1, NFE2, and TFAP4. Moreover, it also had 54 miRNAs (Fig. 8B).

## 4. Discussion

Radiation treatments play an important role in prolonging the survival time of patients with THCA. However, there has been limited research on the radiation effect in THCA patients. Hence, we explored the hub radiation-related prognosis genes and their clinical value and potential mechanisms of action via various bioinformatics based on raw message RNA and long noncoding RNA expression data. In this study, we found that LRP1B and lncRNA AL356596.1 can be independent prognostic factors in THCA patients with radiational treatment via a series of analyses. Compared with the non-chemotherapy group, both genes were lowly expressed in chemotherapy patients.

LRP1B, low-density lipoprotein receptor-related protein 1B, is a member of the low-density lipoprotein receptor protein family. LRP1B is frequently absent or its expression is frequently silenced in various tumor types.^[8–10]^ Many kinds of research indicated that it had different various biological functions in cancer progression and tumor malignancy in different cancer. The reported study showed that it is a tumor suppressor in non-small cell lung cancer.^[8]^ Moreover, the activity of LRP1B is commonly down-regulated during the process of the development of many kinds of cancer, for instance, renal and colon cancer. Ni et al showed that down expression of LRP1B was conducive to tumor metastasis via RhoA/Cdc42 pathway and actin cytoskeleton remodeling in renal cell cancer.^[11]^ Tabouret et al indicated that there was a relationship between the short survival time of glioblastoma and the knockdown of the LRP1B.^[12]^ In the study of H Prazeres et al, it was found that compared with healthy people, the expression of this gene was significantly reduced in thyroid cancer lesions or thyroid cancer, leading to changes in the tumor environment and promoting the growth and invasion of tumor cells.^[13]^ In this work, we found that the expression of LRP1B was also higher in normal than in the THCA, moreover, the expression of the LRP1B in the THCA patients without radiation treatment was lower than in the THCA patients with radiation treatment. The results indicated that the expression of LRP1B was related to the radiation. Based on the reported research, we speculate that chemotherapy may affect the promotion of the gene.^[13]^ The complete methylation of the CpG island of this gene can inhibit LRP1B expression. The promoter region of the gene, the 5’ end untranslated region (5’ UTR) and the first exon region, has a very high density of CpG sequences that exceeds the mean value by more than 5-fold, which becomes enriched for guanine and cytosine, and is called a CpG island (C represents cytosine, p represents phosphate, and G represents guanine).^[14]^ In addition, DNA methylation combined with histone deacetylation also silences the expression of LRP1B.^[15,16]^ We also found that low expression in THCA patients with chemotherapy was beneficial to the prognosis of patients. These results suggested that chemotherapy inhibited its expression, which was beneficial to the prognosis of patients. However, in the process of chemotherapy, how LRP1B inhibits tumor development is unclear and needs further study.

Long noncoding RNAs (lncRNAs) have a very vital function in the development of tumors via various pathways.^[17]^ For example, Cao et al found that LINC02454 promoted the progression of THCA via upregulating HMGA2 through CREB1.^[18]^ Qi et al revealed that the long noncoding RNACATIP-AS1 promoted the progression and metastasis of THCA via the EMT pathway partly by regulating the miR-515-5p and Smad4 expression in THCA cells.^[19]^ In this work, lncRNA AL356596.1 also served as a prognosis-related biomarker. Its expression in tumor tissue was higher than in normal tissue, and its expression was unaffected by radiotherapy. But its expression was also related to the overall survival time and its low expression was helpful for the prognosis of patients with THCA. Our results also found that its expression was inversely proportional to the THCA differentiation score. We speculate that its low expression may promote the differentiation process of THCA, which is beneficial to the prognosis of patients. This speculation needs further study in the future.

GSEA analysis showed that the mTOR signaling pathway and regulation of autophagy were enriched in the high-risk score group. (mTOR) is an important cellular energy metabolism signaling pathway. This pathway integrates energy, nutrient, and growth factor signals from inside and outside the cell, participates in the regulation of cell growth, proliferation, metabolism, and survival, and is a central regulator of cell growth.^[20,21]^ It acts as a central regulatory point in autophagy central regulating point in autophagy.^[22]^ The 2 pathways are related to the immune. Hence immune analyses were performed. The mammalian TORC1 (mTORC1) complex is composed of mammalian target of rapamycin (mTOR), mammalian lethal sec13 protein 8 (mLST8), and mTORC1 regulation-related protein (Raptor). mTORC1 is activated by growth factors and nutrients. mTORC1 and mTORC2 are frequently activated in tumor cells and play important regulatory roles in the tumor microenvironment.^[23]^ We found that the T cell regulatory in the low-risk score group were higher than in the high-risk score. Because activated of mTOR leads to increased regulatory T cell inhibition in vitro via reducing the pro-inflammatory such as TGF-β.^[24,25]^

## 5. Limitation

The study still has some limitations. Firstly, we did not consider the time and dose of chemotherapy due to the lack of data. Secondly, we did not perform external data validation due to the inability to obtain chemotherapy-related data sets. Thirdly, we did not carry out experimental verification conclusions due to the limitations of realistic conditions.

## 6. Conclusion

To conclude, this study suggests that LRP1B is a vital gene that executes function via a variety of pathways in THCA patients with radiotherapy. Radiotherapy could reduce the expression of LRP1B and AL356596.1 and is helpful for prolonging the survival time of THCA patients. Moreover, the nomogram was constructed based on hub radiotherapy-related genes, long noncoding RNA, and clinical features. Moreover, it had a great function in predicting survival time for patients.

## Author contributions

**Conceptualization:** Chun-Tao Liao, Xing-Feng Tu.

**Data curation:** Chun-Tao Liao, Xing-Feng Tu, Guo-Liang Lin, De-Jie Zhang, Ming Zhang.

**Formal analysis:** Chun-Tao Liao, Xing-Feng Tu, Guo-Liang Lin, Peng-Fei Li.

**Investigation:** De-Jie Zhang.

**Methodology:** Chun-Tao Liao, Peng-Fei Li, Ming Zhang.

**Writing – original draft:** Chun-Tao Liao, Xing-Feng Tu, Guo-Liang Lin, De-Jie Zhang, Peng-Fei Li.

**Writing – review & editing:** Ming Zhang.
